# Semi-upright position improves ventilation and oxygenation in mechanically ventilated intensive care patients

**DOI:** 10.1186/cc13448

**Published:** 2014-03-17

**Authors:** F Van Beers, P Vos

**Affiliations:** 1 St Elisabeth Hospital, Tilburg, the Netherlands

## Introduction

A semi-upright position (45° position) in ventilated patients is recommended to prevent ventilator-associated pneumonia (VAP) and is one of the first steps in progressive early mobility. We studied ventilated intensive care patients in a semi-upright position compared with a supine position to explore whether there was an improvement of oxygenation and ventilation.

## Methods

We retrospectively studied 60 patients in a mixed medical, surgical, neurological ICU during 2003 and 2007 [1-3]. In this study the effects of 45° position on the peripheral oxygen saturation (SpO_2_) and the end-tidal carbon dioxide (ETCO_2_) were measured. Body position was changed with a Total Care^® ^bed (Hill-Rom) after results for the supine position (10°) were obtained. Half an hour after the body position was changed, measurements were taken, which included SpO_2 _and ETCO_2_. The results of body position change in the individual patients were analysed with paired-samples t test. A significance level <0.05 was considered significant.

## Results

Mean SpO_2 _supine was 96.55% ± SD 2.404 versus mean SpO_2 _semi-upright 97.4% ± SD 2.423, and mean ET-CO_2 _supine was 4.62% ± SD 1.988 versus mean ET-CO_2 _semi-upright 4.31% ± SD 1.060. The SpO_2 _(*P *< 0.0001) and the ETCO_2 _(*P *< 0.0001) improved significantly for the 45° position compared with <10° position.

## Conclusion

We demonstrated a significant increase in oxygen saturation and a significant decrease in end-tidal CO_2 _when the head of the bed was elevated during mechanical ventilation. We believe positional therapy in intensive care patients is very important. The semi-upright position is an easy, effective and safe treatment in ICU patients. This position is effective in the bundle prevention of VAP, is the first step in progressive early mobility and is also effective in oxygenation and ventilation in mechanically ventilated patients. This clinical benefit of head-of-bed elevation >30° must lead to a standard of care in mechanically ventilated patients. Since 2009 the semi-upright position is a standard of care in our hospital.
